# Autophagy Activation in Zebrafish Heart Regeneration

**DOI:** 10.1038/s41598-020-59106-z

**Published:** 2020-02-10

**Authors:** Myra N. Chávez, Rodrigo A. Morales, Camila López-Crisosto, Juan Carlos Roa, Miguel L. Allende, Sergio Lavandero

**Affiliations:** 1 Advanced Center for Chronic Diseases (ACCDiS) & Corporación Centro de Estudios Científicos de las Enfermedades Crónicas (CECEC), Faculty of Chemical and Pharmaceutical Sciences & Faculty of Medicine, Universidad de Chile, Santiago, Chile; 2 Center for Genome Regulation (CGR), Department of Biology, Faculty of Sciences, University of Chile, Santiago, Chile; 3 Department of Pathology, School of Medicine, Pontificia Universidad Católica de Chile, Santiago, Chile; 4 Department of Internal Medicine (Cardiology Division), University of Texas Southwestern Medical Center, Dallas, USA

**Keywords:** Cardiac regeneration, Experimental models of disease

## Abstract

Autophagy is an evolutionarily conserved process that plays a key role in the maintenance of overall cellular health. While it has been suggested that autophagy may elicit cardioprotective and pro-survival modulating functions, excessive activation of autophagy can also be detrimental. In this regard, the zebrafish is considered a hallmark model for vertebrate regeneration, since contrary to adult mammals, it is able to faithfully regenerate cardiac tissue. Interestingly, the role that autophagy may play in zebrafish heart regeneration has not been studied yet. In the present work, we hypothesize that, in the context of a well-established injury model of ventricular apex resection, autophagy plays a critical role during cardiac regeneration and its regulation can directly affect the zebrafish regenerative potential. We studied the autophagy events occurring upon injury using electron microscopy, *in vivo* tracking of autophagy markers, and protein analysis. Additionally, using pharmacological tools, we investigated how rapamycin, an inducer of autophagy, affects regeneration relevant processes. Our results show that a tightly regulated autophagic response is triggered upon injury and during the early stages of the regeneration process. Furthermore, treatment with rapamycin caused an impairment in the cardiac regeneration outcome. These findings are reminiscent of the pathophysiological description of an injured human heart and hence put forward the zebrafish as a model to study the poorly understood double-sword effect that autophagy has in cardiac homeostasis.

## Introduction

Autophagy is an evolutionarily conserved self-degradation process that involves the engulfment, degradation and recycling of dysfunctional or damaged cellular components^1^. It assumes key roles during adaptation to nutrient starvation^1,2^ elimination of pathogens^3^, while it is fundamental in cell differentiation and tissue patterning during development^4^ and regeneration^5–7^. In the heart, autophagy has been found to be essential for cardiac development, morphogenesis and cardiomyocyte differentiation^8–10^, and is recognized as a highly relevant housekeeping mechanism for the maintenance of the post-mitotic, essentially unreplaceable, cardiomyocytes that constitute it^11^. On the other hand, autophagy has been found to be involved in the onset and progression of many cardiovascular diseases, such as myocardial infarction and chronic heart failure, where it is a riddle how autophagy may have a protective role, while at the same time, aggravating cardiac dysfunction^12,13^. Because of this, autophagy has been recognized as a potential therapeutic target for cardiovascular diseases, though finding the right strategy to use autophagy activation to improve clinical outcome has not been possible until now^14,15^.

The zebrafish (*Danio rerio*) has become a popular vertebrate model organism because of its superb regenerative capacities. Experimental models of heart amputation^16^, cardiomyocyte-specific genetic ablation^17^, hypoxic damage and ischemia/reperfusion injury^18,19^ have all shown that, contrary to adult mammals, the zebrafish is capable of restoring damaged and lost myocardial tissue regardless of its age^20,21^. This regenerative potential has been attributed to the ability of dedifferentiation and robust proliferation of its cardiomyocytes upon injury^22–24^. In addition, the zebrafish has become a useful model organism to study the function and mechanisms of autophagy in the context of development and inflammation^25^. Furthermore, autophagy has been found to be important in zebrafish fin^26^ and skeletal muscle^27–29^ regeneration, while very recent studies have proven the zebrafish as a unique and useful model to study autophagy-related cardiomyopathies and their association to the mechanistic target of rapamycin (mTOR) signaling pathway^30–32^. However, the role that autophagy plays during cardiac tissue regeneration remains to be explored. In the present study, we introduce the use of the zebrafish as a model organism to study the contribution and regulation of autophagy during cardiac regeneration.

## Results

**Figure 1:**
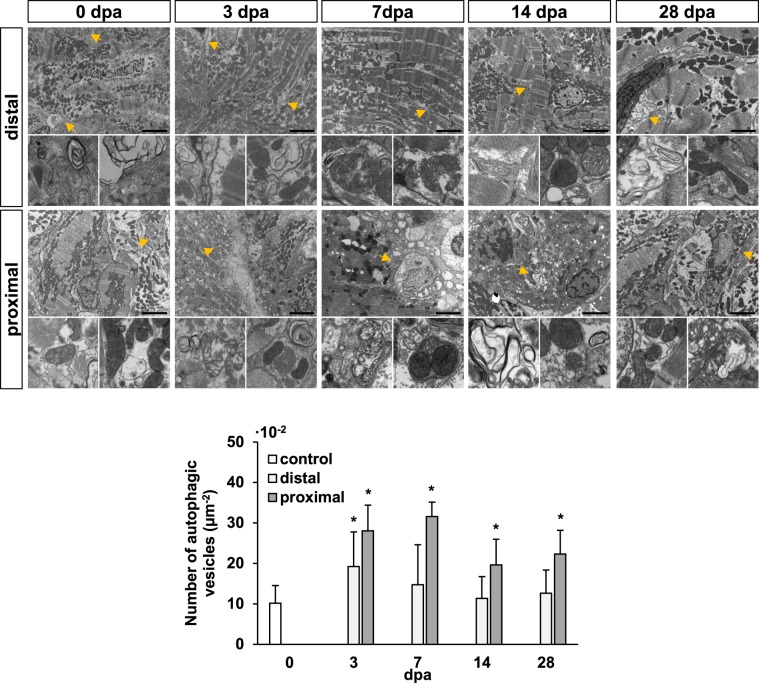
Autophagic vesicle formation in the heart ventricle is increased during zebrafish cardiac regeneration. Electron microscopy shows a high degree of tissue remodeling of the cardiac muscle, as well as the formation of several autophagosome and phagolysosome vesicles upon apex amputation (yellow arrow heads and higher-magnification images below). An increase in the number of autophagic vesicles was above all evident in regions closer to the injured area (proximal), while also found significantly more abundant at 3 dpa distal to the amputation plane. Insets represent higher magnifications of the observed autophagic vesicles at each experimental time point. Scale bars represent 2 μm. *p ≤ 0.05. N ≥ 3.

With the aim of studying the importance of autophagy during zebrafish cardiac regeneration, we investigated the autophagy-related events in the context of the ventricle apex amputation model. In this experimental paradigm, 20% of the zebrafish heart ventricle is surgically removed, and due to its cardiac regeneration capacity, tissue restoration is completed in about 40–60 days^16^. Our electron microscopy data showed a high degree of tissue-remodeling of the cardiac muscle above all between 3 and 14 days post-amputation (dpa). Moreover, several autophagosomes and phagolysosome vesicles were observed, in particular in regions proximal to the injured area (Fig. 1), where their number was found significantly higher when compared to the uninjured heart. Interestingly, a significant increase in autophagic vesicles was also observed at 3 dpa in distal areas of the ventricle. Cardiac tissue seemed to return to its original morphology during the regeneration process and fewer autophagic vacuoles were found at 28 dpa, though still significantly higher represented in number.

**Figure 2:**
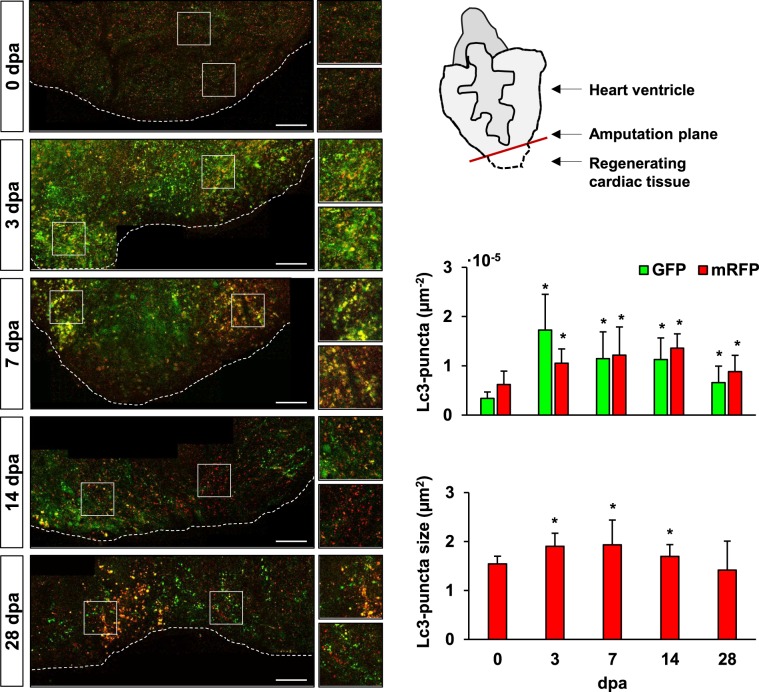
Enhanced accumulation of autophagosomes and phagolysosomes is observed in the injured cardiac tissue upon amputation. The transgenic line *Tg(b-actin2:mRFP-GFP-Lc3)* allows the observation of autophagosome formation and processing in the regenerating zebrafish heart ventricle at different time points after apex amputation. Autophagosomes are depicted both as GFP- and RFP-puncta, while the lower pH-sensitivity of RFP additionally allows the visualization of phagolysosomes. Images were taken immediately after heart excision, and upon overnight incubation of the zebrafish in 2 mM CQ, which acts as a lysosomal inhibitor. Quantitative analysis revealed a significant increase in the number of both vesicles, as well as in the average autophagosome/phagolysosome size (red puncta) in the regenerating tissue, which gradually decreased over time. A schematic view of the ventricle orientation and the region of interest considered for image acquisition is shown, while higher magnifications exemplifying the accumulation of bigger autophagic vesicles are presented next to the mosaic images. Scale bars represent 200 μm. *p ≤ 0.05. N ≥ 3.

In order to study the dynamics of autophagy, we generated a transgenic zebrafish line to observe autophagosome formation and processing in the regenerating heart at different time points after amputation (Fig. 2). *Tg(b-actin2:mRFP-GFP-Lc3)* zebrafish ubiquitously express the autophagosome-building microtubule-associated protein 1 A/1B light chain 3B (Lc3), which is linked to both red and green fluorescent proteins (mRFP, GFP). In basal conditions, this protein is uniformly distributed in the cytoplasm, however when autophagy is activated, mRFP-GFP-Lc3 is recruited to the phagosome-membrane which generates quantifiable discrete fluorescent signals. Furthermore, due to specific sensitivity of GFP to low-pH, it is possible to follow lysosomal degradation of the autophagosomes, where early autophagosomes are observed as GFP- and RFP-puncta, while phagolysosomes are only labelled with RFP. Treatment with the lysosomal-inhibitor chloroquine (CQ) allowed the observation of a significant accumulation of GFP and mRFP positive autophagosomes near the injured area, which peaked at 3 dpa and slowly decreased over time (Fig. 2). While GFP:mRFP ratios were significant at 3 dpa and 7 dpa compared to uninjured hearts (0 dpa 0.62 ± 0.35, 3 dpa 1.66 ± 0.56, 7 dpa 1.08 ± 0.49, p ≤ 0.01), the number of mRFP-puncta peaked later, reflecting the dynamics of autophagosome-processing. Also, phagolysosome size (mRFP^+^ puncta) increased significantly between 3–14 dpa, and then returned to normal by 28 dpa (Fig. 2).

**Figure 3:**
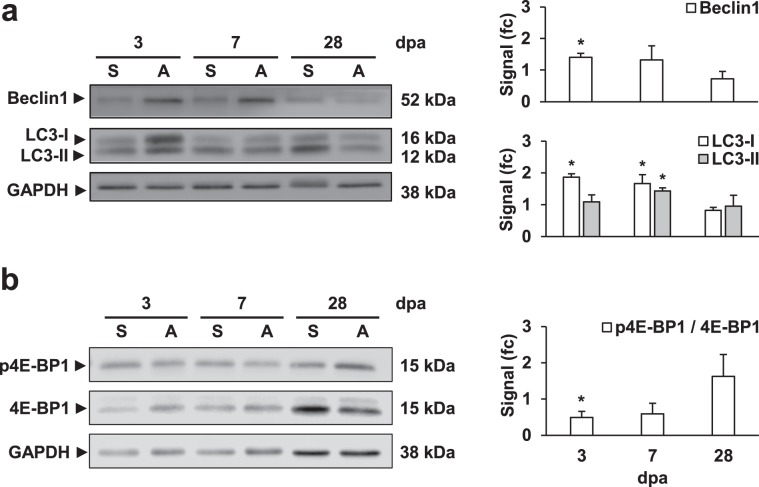
Western blot analysis suggests the activation of autophagy at the early stages of cardiac regeneration. The stimulation of autophagy at 3 dpa and the increased formation of autophagosomes between days 3–7 post-amputation, in comparison to sham-operated animals, is observed in the increased abundance of Beclin1, Lc3-I and Lc3-II, respectively (**a**). Moreover, a significant decrease on the phosphorylation ratio of the mTOR-target 4E-BP1 suggests the inhibition of mTOR at the early regeneration stages (**b**). Signal levels were normalized first to GAPDH-loading controls and then compared to the respective sham-control. Results were obtained from at least three biological replicates and averaged for the final statistical analysis. Representative blot-images of are shown, while full-length blots are presented in the Supplementary Information (Supp. Fig. 1). fc: fold change. *p ≤ 0.05. N ≥ 3.

Lastly, we were able to determine an increased presence of the autophagy promotor protein Beclin1 at 3 dpa by using Western blot analysis (Fig. 3a). Also, the levels of Lc3-I and II were higher to those in sham-operated controls at 3 and 7 dpa (Fig. 3a), while the ratio Lc3-II/I was significantly higher at 3 dpa (sham 1.13 ± 0.12, amputated 0.66 ± 0.78, p ≤ 0.05), indicating an enrichment of Lc3-I, which was later seen as an increased Lc3-II at 7 dpa. Moreover, an inhibition in mTOR-signaling was observed at 3 dpa based on the significant decreased phosphorylation of the mTOR-target 4E-BP1 compared to the sham-control (Fig. 3b). Protein signals did not vary significantly between amputated and control samples at 28 dpa, thereby suggesting that by then, autophagy had returned to basal levels.

**Figure 4:**
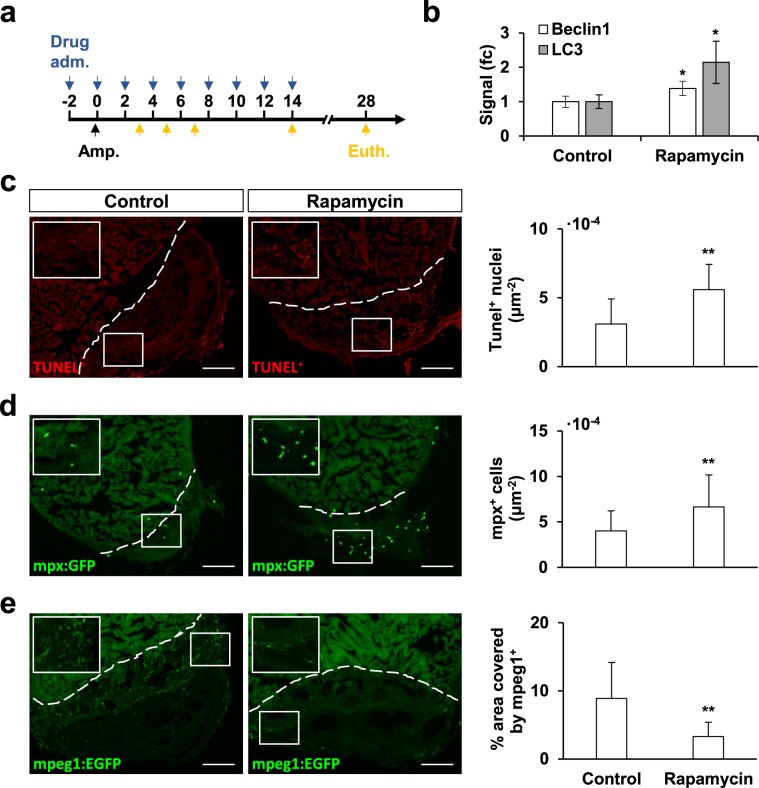
Rapamycin treatment affects the inflammatory phase of the cardiac regeneration process. Zebrafish were treated with rapamycin via intraperitoneal injection two days before the amputation procedure and every second day afterwards (**a**). The effect of rapamycin treatment was verified by the accumulation of Beclin-1 and Lc3 after 9 doses. Results were obtained from at least three biological replicates. Signal levels were normalized first to GAPDH-loading controls and then compared to the control treatment. (fc: fold change). Full-length blots representative of the results can be found in the Supplementary Information (Supp. Fig. 3). Upon amputation, rapamycin-treated zebrafish showed an increased number of apoptotic TUNEL^+^-nuclei in the affected ventricle area (**c**), as well as an increased presence of mpx^+^-neutrophils (**d**). Also, mpeg1^+^-macrophage recruitment at 5 dpa was affected by the treatment, as shown by their decreased presence of the affected area (**e**, % area covered by macrophages). Altogether, the results suggest an alteration in the inflammatory mechanisms triggered upon apex amputation. The results obtained from the quantification of all fluorescent signals were normalized to the area of the regenerating tissue considered, which was delimited by the amputation plane on the ventricle (white discontinuous line). Scale bars represent 50 μm. TUNEL^+^: Control N ≥ 14, Rapamycin N ≥ 13; mpx^+^: Control N ≥ 14, Rapamycin N ≥ 16; mpeg1^+^: Control N ≥ 8, Rapamycin N ≥ 9. *p ≤ 0.05, **p ≤ 0.001.

To investigate how sustained autophagy could affect the regeneration process, autophagy was induced via intraperitoneal injection of the mTOR-pharmacological inhibitor rapamycin (Fig. 4a). We showed that rapamycin administration induced an enrichment of Beclin1 and Lc3 in the zebrafish heart after 9 doses, which corresponded to 14 dpa in operated zebrafish (Fig. 4b, Supp. Fig. 3); yet, contrary to what was expected, a significant increase in the phosphorylation levels of 4E-BP1 was observed as a consequence to rapamycin treatment (Supp. Fig. 4). No significant weight-loss was observed as a consequence of the pharmacological treatment (mean weight at 0 dpt: 490 ± 62 mg, mean weight at 14 dpt: 480 ± 45 mg, N ≥ 6). However, because of rapamycin treatment, apoptosis-rate in the injured tissue (Fig. 4c) and presence of mpx^+^-neutrophils (Fig. 4d) were significantly increased at 3 dpa. In addition, the number of mpeg1^+^-macrophages was significantly diminished compared to control animals (Fig. 4e). All these observations suggest that the excessive activation of autophagy in the zebrafish heart had a direct effect in the inflammatory processes triggered upon amputation.

**Figure 5:**
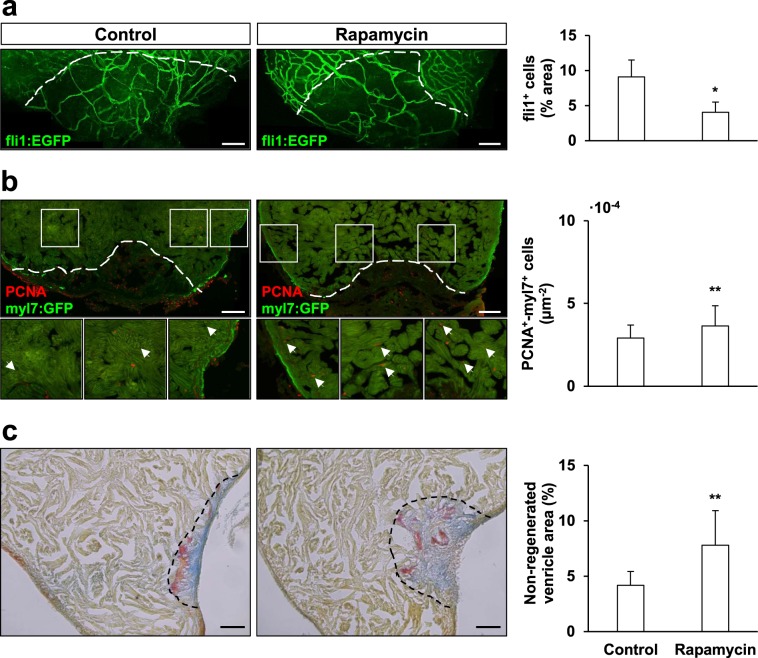
Rapamycin treatment affects zebrafish cardiac regeneration. Rapamycin-treatment affected angiogenesis upon amputation, causing a negative effect in the re-vascularization rate of the regenerating zebrafish ventricle at 7 dpa (**a**, fli1a^+^-covered area expressed in relation to the regenerating ventricle area); yet, the same treatment had a specific positive effect over cardiomyocyte proliferation at 14 dpa (normalized by the area considered for the quantification) (**b**). Still, sustained rapamycin administration impaired zebrafish cardiac regeneration, as shown by the AFOG-staining of amputated treated and control hearts at 28 dpa, which allows to visualize the remains of fibrin (red)- and collagen (blue)- provisional extracellular matrices, as well as the uninjured/restored cardiac muscle (yellow/brown) (**c**). The quantification of the non-regenerated area (normalized to the total ventricle area) showed that treatment with rapamycin for up to 14 dpa caused a significant impairment of the regenerative outcome of the zebrafish heart. Scale bar represents 100 µm in (**a**,**b**) and 50 µm in (**c**). fli1a^+^: Control N ≥ 8, Rapamycin N ≥ 7; PCNA^+^-myl7^+^: Control N ≥ 8, Rapamycin N ≥ 9, AFOG-staining: Control N ≥ 10, Rapamycin N ≥ 12. *p ≤ 0.05, **p ≤ 0.001.

We next decided to study whether sustained autophagy could also affect later regeneration relevant processes such as angiogenesis and cardiomyocyte proliferation. Here, we observed a significant impairment in the re-vascularization of the injured ventricle area of rapamycin-treated fish at 7 dpa (Fig. 5a). Interestingly, rapamycin treatment had a specific positive effect over cardiomyocyte proliferation at 14 dpa (Fig. 5b), where we found that, while the overall number of PCNA^+^ nuclei did not change upon rapamycin treatment (data not shown), the number of PCNA^+^/myl7^+^ cardiomyocytes was significantly increased.

Finally, we compared the regeneration progress in rapamycin treated zebrafish against control zebrafish at 28 dpa (Fig. 5c). AFOG-staining of heart cryosections allowed to observe the remains of fibrin (red) and collagen (blue) provisional extracellular matrices that are deposited upon injury, as well as the uninjured and regenerated cardiac muscle (yellow-brown). Our results showed that the non-regenerated ventricle area was significantly larger in rapamycin-treated zebrafish compared to control animals, hence suggesting that sustained autophagy activation might impair zebrafish heart regeneration.

## Discussion

Heart failure is regarded a global pandemic that affects about 26 million people around the globe^33^. It is therefore a priority to find novel therapeutic approaches that protect the cardiac tissue, improve the healing outcome, and prevent the pathological progression from an acute myocardial injury to a chronic disease^14,15^. In this regard, autophagy is a homeostatic process that can be activated to prevent cell death and provide metabolic substrates under stress conditions. Given the regenerative capacity of the zebrafish heart, in this study, we introduce the zebrafish as a model that could be useful to understand how autophagy is regulated in the context of a regenerating tissue.

Our results suggest that, indeed, autophagy is activated during the early inflammatory stage after heart amputation (Fig. 1). And, as in the tail fin^26^, skeletal muscle^27–29^, and infarcted murine heart^34^, increased autophagosome formation is observed under electron microscopy in the cardiac ventricle tissue near the injury margin, and is gradually downregulated to basal levels as regeneration progresses. Moreover, making use of the generated transgenic line we were capable to observe a significant accumulation of the autophagic vesicles within the regenerating tissue. This experiment required the overnight incubation of the zebrafish in 2 mM CQ (Fig. 2), in order to allow the detection of the discrete quantifiable LC3-mRFP-GFP signals. Thus, the results obtained led us to propose that the increased number of autophagic vesicles comes as a result of autophagy activation, and not because of autophagy impairment. However, autophagic flux in the presence and absence of lysosomal inhibitors should be studied at each regeneration stage to clarify this paradigm. Also, the abundance of the autophagy markers Beclin1 and Lc3-I/II was found to be increased in amputated ventricles compared to sham-operated ventricles, thus suggesting that changes in the autophagic response are triggered by the regeneration process, and not as a consequence of the inflammation caused by the surgical procedure.

mTOR is a serine/threonine protein kinase, which upon binding to the regulatory subunits Raptor and mLST8, forms the mTORC1 complex. mTORC1 regulates several biological processes by acting as a nutrient sensor, while in turn promoting cell growth, proliferation, and survival. In particular under (basal) nutrient-rich conditions, mTORC1 acts as a negative regulator of autophagy^35^. In this regard, the significant decrease of the mTOR phosphorylation substrate 4E-BP1 observed at 3 dpa could indicate a link between the stimulation of autophagy upon injury and the inhibition of mTOR-signaling.

In order to explore the role for this stereotyped autophagy response after heart injury, we decided to alter autophagy activation by pharmacological means and to investigate how the injured cardiac tissue responded under such circumstances. Rapamycin is known to specifically bind to mTORC1 and inhibit its downstream signaling. In line with this, treatment with rapamycin has been shown to induce autophagy in several experimental settings, and thus is considered a standard positive control in autophagy research^36^. In this study, the sustained accumulation of the autophagy marker-proteins Lc3 and Beclin1 in the zebrafish heart was achieved by intraperitoneal administration of rapamycin for 14 days, when compared to a control treatment (Fig. 4b). However, protein analysis also revealed a significant increase in the phosphorylation state of 4E-BP1 as a consequence to rapamycin treatment (Supp. Fig. 4). According to the literature, prolonged rapamycin treatment may lead to hyperphosphorylation of 4E-BP1 in a cell-specific manner^37^. Moreover, other studies have shown that some 4E-BP1 phosphorylation sites are more insensible towards rapamycin, and hence advise other targets, such as the ribosomal S6 kinases (e.g. 70S6K), to address mTOR-inhibition^38^. Unfortunately, the phosphorylated 70S6K could not be detected in our samples (data not shown), and thus it was not possible to conclusively demonstrate the complete inhibition of mTOR.

Nevertheless, we observed that treatment with rapamycin had a significant effect over apoptosis and immune cell recruitment at 3 and 5 dpa in the regenerating tissue (Fig. 4). These time points were chosen according to the reported apoptosis and inflammatory cell-recruitment maxima^39,40^ in the zebrafish model, where it has been described that altered presence of inflammatory cells as a result of decreased pro-inflammatory neutrophil clearance or a diminished macrophage recruitment response, have a direct repercussion in the regeneration outcome^41–43^. Interestingly, modulating inflammatory therapeutic strategies are being considered as an alternative option to minimize the damage upon a myocardial infarct^44,45^. On the other hand, while early activation of autophagy upon injury is supposed to function as a cardioprotective mechanism that controls the extent of cell-death, it is also known that excessive autophagy may result in the elimination of essential cellular constituents, thereby promoting cell-death^11^. In this regard, the apoptosis-rate in the zebrafish apex amputation model is reported to be very low^46^, yet it was significantly incremented in the rapamycin-treated group (this study). These results suggest that, in our model, it possible to study how autophagy is capable of stimulating both inflammation and cell death, which is known to worsen cardiac tissue damage^47,48^. Moreover, we found out that revascularization was impaired in rapamycin-treated zebrafish, where a deficit in the ability to grow and extend new vessels towards the injured area was observed (Fig. 5a). Revascularization of the regenerating ventricle has been shown to be essential for correct tissue restoration in both zebrafish^49,50^ and murine models^51^, while several studies have shown that autophagy regulation is necessary for the proper adaptive response of the vascular system^12^.

As mentioned before, the zebrafish cardiac regeneration potential relies on the proliferative capacity of its cardiomyocytes, which peaks at 14 days post-amputation, as previously shown by the assessment of nuclear incorporation of bromodeoxyuridine (BrdU)^16^. In our experimental setup, we showed that treatment with rapamycin up to this stage significantly increased the proliferation of cardiomyocytes (Fig. 5b). While this result may seem surprising, it has been described that cardiomyocyte proliferation is elevated in anemic zebrafish during regeneration^18^. Moreover, there have been reports of increased proliferation in the heart muscle of mTOR-KO mice^52^, while PPARδ, a regulator of energy homeostasis, has been found to promote both mammalian and zebrafish cardiomyocyte proliferation^53^. Interestingly, a recent review article summarizing novel therapeutic strategies to mitigate heart failure concluded that combining anti-apoptosis approaches targeting post-infarction granulation tissue with cardiomyocyte-specific pro-autophagy therapies would lead to the best clinical outcome^54^. The above result notwithstanding, at 28 dpa the degree of heart ventricle regeneration was significantly poorer in the rapamycin-treated group than in the control animals, since the former showed a larger ventricle area occupied by collagen, a marker of non-regenerated tissue. On this subject, altered autophagy is regarded as a hallmark of diseased and failing hearts, while it is known that the pathophysiological mechanisms leading to chronic heart failure are associated with cardiac tissue remodeling along with collagen deposition^55,56^.

Our study leaves unsolved whether sustained autophagy activation is capable of completely impairing the regenerative capacity of the zebrafish heart, or if it only causes a delay. Furthermore, future studies should address if cardiac function restoration is affected by sustained autophagy activation. In addition, while we chose the ventricle apex amputation model, because it is very well-characterized, and allows to easily follow the restoration of lost cardiac tissue, other models could be implemented to study the role that autophagy plays in heart failure^57^, cardiomyocyte hypertrophy^58,59^, ischemia/reperfusion injury^18,19^ or in the context of a fibrotic response caused by a cryoinjury^60,61^, which resemble the tissue damage upon myocardial infarction in the human heart.

On a critical note, the experiments performed do not allow to discriminate if the differences observed upon rapamycin-induced autophagy are unrelated to other processes for which mTORC1 is responsible for, such as protein synthesis and cellular growth^62^. In this regard, it would be key to analyze the status of mTOR signaling and all of its targets along the course of the regeneration, as well as to investigate the effect of mTORC1-independent autophagy-inducer mechanisms such as the AMPK- and GSK-3β-dependent signaling pathways, which have been found relevant for the survival of ischemic cardiomyocytes^63^. Moreover, in order to understand the importance of this homeostatic process and to demonstrate causality between increased autophagic activity and defective cardiac regeneration, future studies are required to address these points by using genetic models of defective autophagy and/or pharmacological inhibitors. Also, to comprehend the role that autophagy plays in the early stages of cardiac repair and its exact function, it would be necessary to elucidate both the cell-specificity of the autophagic response, as well as the precise signaling pathways that activate it upon damage. Plus, given the relevance of the activation of specific cardiac tissues such as the epicardium, endocardium, and endothelium for the orchestration of the regeneration process^64–66^, it would be intriguing to know if autophagy modulation is of particular relevance for them.

Understanding how autophagy modulation helps to promote myocardial regeneration may lead to alternative therapeutic targets for humans to assist in myocardial infarction recovery and the treatment of other chronic cardiovascular diseases. Since the current availability of genetic tools are allowing the zebrafish to become an advantageous model for the study of autophagy^25,67^, future studies will be able to address the precise mechanisms and consequences of autophagy activation upon cardiac injury based on it.

## Materials and Methods

### Zebrafish husbandry and animal operations

Adult zebrafish (*Danio rerio*) from the wild type Tab-strain or transgenic strains *TgBAC(mpx:GFP)i114*^68^*, Tg(mpeg1:EGFP)*^69^, *Tg(fli1a:EGFP)y141*^70^ and *Tg(myl7:GFP)*^71^ were kept and grown in our animal facility. Zebrafish were maintained under monitored water conditions (28.0 °C, pH 7.0–7.3, 600–800 μS) in 14:10 light-dark cycle conditions at a density of 2–4 fish/L and daily fed with dry particularized food (Gemma, Skretting, Norway). Animals used within an independent experiment were siblings grown in the same tank and were randomly divided into the experimental groups. All animal procedures were performed under anesthesia with MS-222 (Tricaine, A5040, Sigma-Aldrich, MO, USA). Surgical apex amputation operations were performed according to previous reports^16,72^. Briefly, 6–18 months old zebrafish, are placed on a moist sponge ventral side up. By observing under a stereoscope, scales are removed from the chest area using tweezers, then an incision through the skin and pericardial sac is made to expose the ventricle. The ventricle apex is gently pulled up and using curved vannas scissors approximately 20% is removed. Bleeding is stopped by lightly holding a cotton swab over the wound for some seconds. Once the wound has clotted, fish are returned to water and carefully reanimated by squirting water over the gills with a plastic pipette. Fish were then taken back to the facility for recovery. Euthanasia was performed by overdose of anesthetics. All procedures complied with the “Guidelines for the Use of Fishes in Research Use” of the American Fisheries Society (Guidelines for the use of fishes in research. American Fisheries Society, Bethesda, Maryland. www.fisheries.org) and were approved by the Animal Ethics Committee of the University of Chile.

### Transmission electron microscopy

At 3, 7, 14, and 28 dpa (N ≥ 3) zebrafish were euthanized and regenerating hearts were dissected. Uninjured hearts from sibling zebrafish were used as controls. Samples were fixed in glutaraldehyde, and then rinsed in osmium tetroxide (8% in veronal buffer), dehydrated in acetone gradient, and finally, embedded in EPON^TM^ epoxy resin (Miller-Stephenson, USA). Semi-thin sections were cut and stained with Toluidine Blue to confirm the sample orientations. Afterwards, ultrathin sections were obtained, collected on a cooper grid, and finally stained with Reynolds’ lead citrate solution for electron microscopy (Phillips Tecnai 12 Biotwin, Netherlands). Identification and quantification of the autophagic vesicles observed in the uninjured ventricle, as well as in the proximal (near the ventricular apex/affected area) and distal areas (opposite site of the ventricle) in relation to the amputation plane were performed according to the descriptions and examples found in the literature^25,73^ in at least 5 picture-frames (95 µm^2^) per experimental time point.

### Generation and use of the *Tg(bactin2:mRFP-GFP-Lc3)* transgenic line for analysis of autophagic vesicle formation upon apex amputation

The pDest-bactin2:mRFP-GFP-Lc3 construct was generated following the Gateway® recombination cloning protocol^74^. The middle entry vector pME-tl-zfLc3 was kindly provided by Dr. Angeleen Fleming (University of Cambridge, UK), with authorization of Dr. Tamotsu Yoshimori (Osaka University, Japan) who created the original construct^75,76^. The rest of the constructs used (299-p5E-bactin2, 302-p3E-polyA, 394-pDestTol2pA2) were a gift from Dr. Kristen Kwan (University of Utah, USA). Apex amputation was performed on the transgenic 10–14 months old zebrafish. Then, in order to obtain discrete strong fluorescent signals, control and amputated zebrafish were incubated overnight in 2 mM CQ, which induced the accumulation of the autophagic vesicles. Image acquisition of the injured and control transgenic hearts was performed immediately after excision. For this, ventricles were placed in cold PBS in between coverslips and oriented to allow a transversal view of the injury. The region of interest for image analysis considered an area of at least 0.2 mm^2^ (5 frames, 212.55 mm * 212.55 mm) using the ventricle border as a reference. Quantification of autophagic vesicle numbers and sizes was performed on Z-projections of the images taken using the software ImageJ^77^.

### Western blot analysis

Zebrafish amputated and control-sham ventricles were dissected at 3, 7, and 28 dpa. Pooled protein extracts were obtained from 2–4 ventricles for each experimental group, first by tissue mechanical homogenization in RIPA buffer (10 mM Tris pH 7.2, 150 mM NaCl, 0.1% SDS, 0.5% sodium deoxycholate, 1% Triton-X 100) and then by sonication, and thus considered as a single biological replicate. Denatured samples (5 min, 95 °C, 5% β-mercaptoethanol) were then separated by SDS-PAGE and transferred to PVDF-membranes following standard protocols. Antibodies used for these experiments were anti-LC3 (1:1000; rabbit, #2775S, Cell Signaling, USA), anti-Beclin1 (1:1,000; rabbit, #3738, Cell Signaling, USA), anti-GAPDH (1:5000; goat, #G9545, Merck. Germany), anti-4E-BP1 (1:1000; rabbit, #9644, Cell Signaling, USA), anti-phospho-4E-BP1 (Thr37/46) (1:1000; #2855, Cell Signaling, USA). The enhanced chemiluminescent substrate SuperSignal^TM^ West Femto (Thermo Fisher Scientific, USA) was used for signal detection. Signal intensities were quantified using the software ImageJ^77^, then normalized to their respective loading control (GAPDH), and finally to the signal corresponding to the sham-control condition.

### Pharmacological treatment

Autophagy was induced in zebrafish by injection of the mTOR-inhibitor rapamycin (Rapamycin from *Streptomyces hygroscopicus*, ≥95% (HPLC), Merck, Germany). A 20 mg/ml stock solution was first prepared in DMSO, then a working solution was prepared by diluting the drug to 1 mg/ml in Cortland-solution. Following a described protocol for zebrafish intraperitoneal injection^78^, the fish were placed under anesthesia as described above, and placed on a moist sponge ventral side up. Using a Hamilton syringe, a dose of 5 mg rapamycin/kg body weight was injected every two days according to the experimental design described in Fig. 4a. Control animals were injected 5% DMSO in Cortland-solution. To experimentally prove the pharmacological stimulation of autophagy, the expression of Lc3-I/II and Beclin1 was assessed via Western blot after nine doses of rapamycin. For this, zebrafish were kept in water containing 1 mM chloroquine (Chloroquine diphosphate salt, ≥98%, Merck, Germany) for 3 h before euthanasia in order to trigger autophagosome accumulation. Pooled protein lysates were prepared from 2–3 ventricles, and thus considered as one biological replicate for the Western Blot analysis. Signal intensities were quantified using the software ImageJ^77^, and normalized to the respective loading control (GAPDH).

### Cryosection and acid-fuchsin orange G (AFOG) staining

Zebrafish hearts were dissected and fixed in fish fix (4% (w/v) paraformaldehyde in fish buffer) at room temperature for 1 h, washed three times in fish buffer (4% (w/v) sucrose, 0.12 μM CaCl_2_, 0.077 M Na_2_HPO_4_, 0.23 NaH_2_PO_4_, pH 7.4) and then left in 30% (w/v) sucrose at 4 °C overnight. Hearts were then immersed in OCT (Tissue-Tek, Sakura Finetek, USA) and immediately stored at −80 °C. For AFOG staining, 18 µm cryosections were dried at 37 °C for 1 h, and then incubated at 60 °C for 3 h in Bouin-fixative previously heated at 60 °C for 30 min. Slides were then rinsed several times in distilled water (dH_2_O) for 30 min, and the incubated with 1% (w/v) phosphomolybdic acid solution for 5 min. Following a 5 min wash-step in dH_2_O, sections were stained with the AFOG-solution for 5 min, washed in running water, dehydrated in ethanol series, cleared in xylene, and finally mounted in Entellan® (Merck, Germany). Quantification of the non-regenerated area of the ventricle was done on the three largest collagen-fibrin covered scar areas found for each heart, which was then normalized by the whole ventricle area.

### Immunohistochemistry (IHC) and TUNEL assay

For immunofluorescence, 15 µm cryosections were dried at 37 °C for 1 h, then washed three times with PBS-Tween (1X phosphate buffered saline, 0.1% Tween-20) and then incubated in blocking solution (2% (v/v) horse serum, 10% (v/v) goat serum, 10% (v/v) fetal calf serum, 1% (v/v) DMSO in PBST). For PCNA-IHC, antigen retrieval was performed by boiling samples at 98 °C for 20 min in pre-heated citrate buffer (10 mM citric acid, 0.05% Tween 20, pH 6.0), and allowing them to cool for another 20 min, before the PBS-Tween washing steps. Samples were then incubated in primary antibodies overnight at 4 °C, followed by three PBS-Tween washes and incubation with secondary antibodies for 3 h at room temperature (RT). Samples were washed again and mounted in 70% glycerol. For whole mount IHC, hearts were excised and fixed in paraformaldehyde (4% (wt/vol) in PBS, 1 h, RT), and then washed three times with PBS-T (1x PBS, 0.1% Triton X-100) and twice with dH_2_O. The hearts were next incubated in 3% (vol/vol) H_2_O_2_ in methanol for 1 h for permeabilization before blocking. For Terminal deoxynucleotidyl transferase (TdT) dUTP Nick-End Labeling (TUNEL)-assay was performed according to the manufacturer’s instructions (ApopTag® Red Kit, Merck, Germany). The primary antibodies used in this study were: anti-GFP (1:500, mouse, #MAB3580, Merck, Germany), anti-GFP (1:500, rabbit, #A-11122, Thermo Fisher Scientific, USA), and anti-PCNA (1:250, mouse, #P8825, Merck, Germany), while the secondary antibodies used were: Alexa Fluor® 488 goat anti-mouse, Alexa Fluor® 488 goat anti-rabbit, and Alexa Fluor® 594 goat anti-mouse (1:1,000; Thermo Fisher Scientific, USA).

### Fluorescence microscopy and image analysis

Image acquisition was performed using the following equipment: upright light microscope Leica DM500 (Leica Microsystems, Switzerland), epifluorescence microscope Olympus MVX10 (Olympus Corporation, Japan), and the confocal microscopes Zeiss LSM 510 and Zeiss LSM 710 (Carl Zeiss AG, Germany). Image analysis and quantification were performed using the software ImageJ^77^. In the case of IHC-samples, at least two cryosections containing the most representative areas of the injured/regenerating tissue were considered for each heart ventricle. For the analysis, regions of interest were first digitally selected to cover the entire injured/regenerating tissue starting from the amputation plane. Then, in order to analyze the fluorescens signal, fixed parameters for particle size and shape (TUNEL^+^-nuclei, mpx^+^-cells) and pre-determined signal-thresholds (TUNEL^+^-nuclei, mpx^+^-cells, mpeg1^+^-cells, fli1a^+^-vessels) were selected to allow software-assisted quantification Cardiomyocyte proliferation was determined on blinded-samples, for which descriptions of the specific morphology of the cardiomyocytes and their nuclei^79^ were used as guidelines. For normalization, the results were divided by the area of the region of interest, which considered a margin of 300 µm above the amputation plane and the regenerating tissue beyond.

### Statistical analysis

The results were generated from at least 3 independent experiments. Data is expressed as mean ± SD. For statistical analysis, two-tailed Student’s t-test was used to compare the differences between experimental and control groups and were considered significant when p ≤ 0.05.

## Supplementary information

Supplementary Figures.

## Data Availability

The datasets generated during the current study are available from the corresponding author on reasonable request.
