# Supplementary material for: Autophagy Activation in Zebrafish Heart Regeneration
Source: Sci Rep. 2020 Feb 10;10:2191. doi: 10.1038/s41598-020-59106-z (PMC7010704; doi:10.1038/s41598-020-59106-z)
Supplement: Supplementary file 1 — Supplementary Figures. [file 41598_2020_59106_MOESM1_ESM.pdf]

Autophagy Activation in Zebrafish Heart Regeneration

Myra N. Chavez[a,b], Rodrigo A. Morales[b], Camila López-Crisosto[a], Juan Carlos Roa[c], Miguel L. Allende[b*], Sergio Lavandero[a,d*].

[a]*Advanced Center for Chronic Diseases (ACCDiS) & Center for Molecular Studies of the Cell (CEMC), Faculty of Chemical and Pharmaceutical Sciences & Faculty of Medicine, University of Chile, Santiago, Chile.*
[b]*Center for Genome Regulation (CGR), Department of Biology, Division, Faculty of Sciences, University of Chile, Santiago, Chile.*
[c]*Department of Pathology, School of Medicine, Pontificia Universidad Católica de Chile, Santiago, Chile.*
[d]*Department of Internal Medicine (Cardiology Division), University of Texas Southwestern Medical Center, Dallas, USA.*

***Corresponding authors**

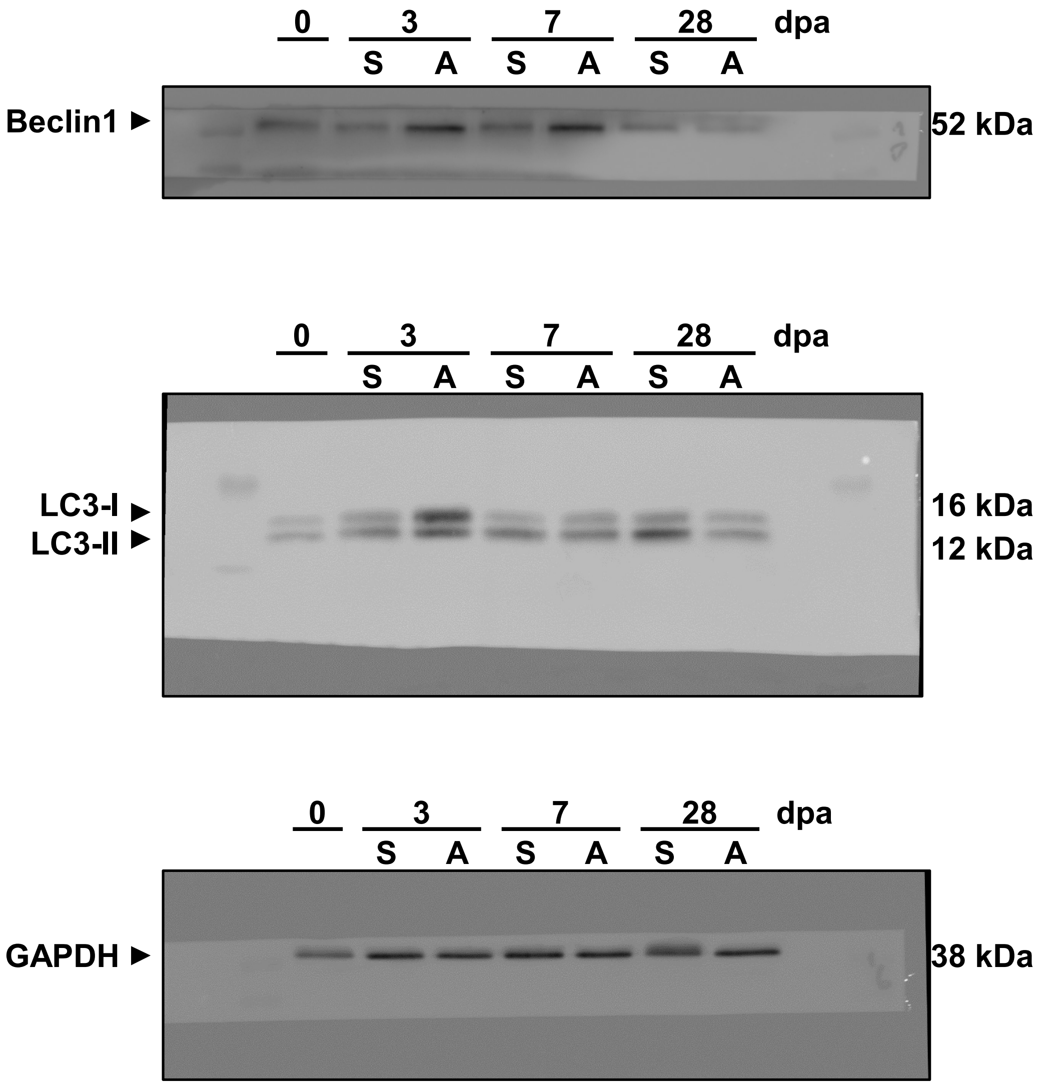

Supp. Fig. 1: Representative full-length blots used in protein analysis of regenerating heart samples. Zebrafish amputated and control-sham ventricles were dissected at 3, 7, and 28 dpa. Pooled protein extracts were obtained from 2-4 ventricles for each experimental group. One pooled protein extract was considered as a biological replicate for data analysis. For quantification, signals were normalized to their respective loading control (GAPDH), and finally compared to the signal of its respective sham-control condition. N≥3.

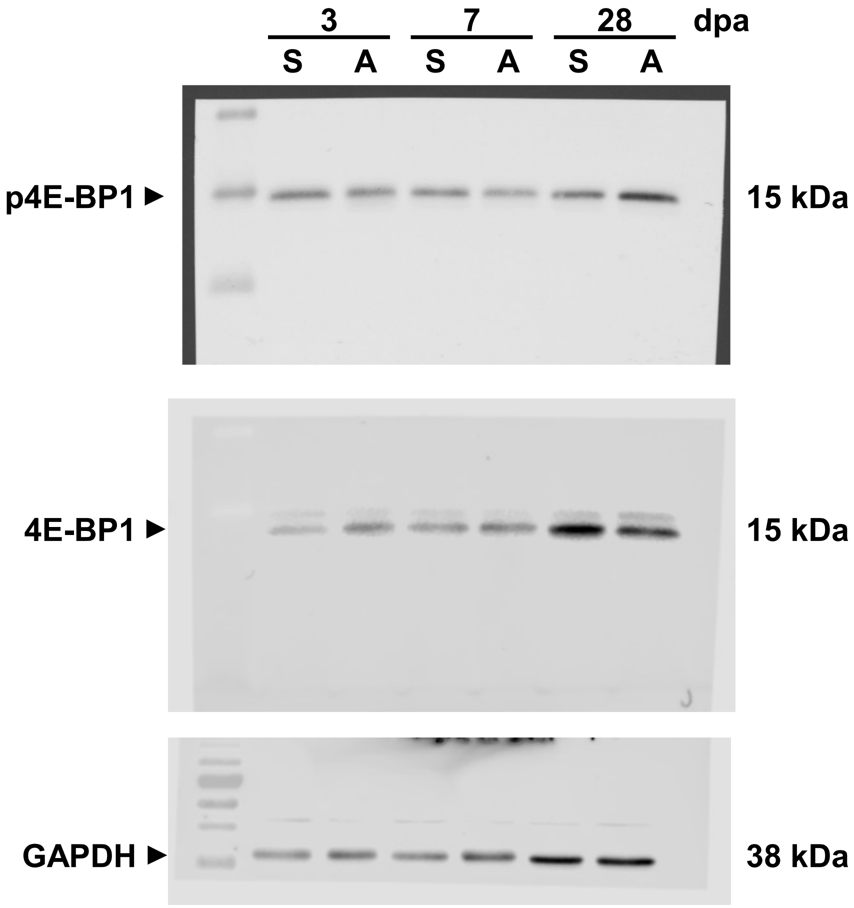

Supp. Fig. 2: Representative full-length blots used in protein analysis of mTOR-signaling in regenerating heart samples. Zebrafish amputated and control-sham ventricles were dissected at 3, 7, and 28 dpa. Pooled protein extracts were obtained from 2-4 ventricles for each experimental group. One pooled protein extract was considered as a biological replicate for data analysis. For quantification, signals were normalized to their respective loading control (GAPDH), and finally compared to the signal of its respective sham-control condition. Finally, a ratio between the phosphorylated and unphosphorylated form of 4E-BP1 was calculated. N≥3.

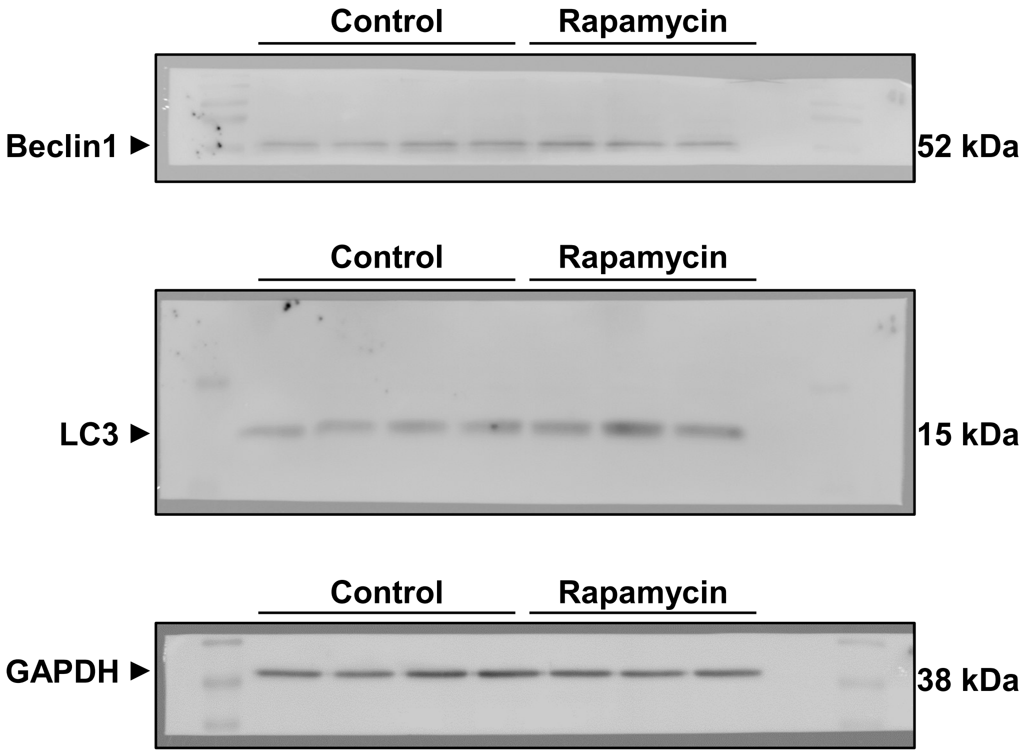

Supp. Fig. 3: Full-length blots used in protein analysis of pharmacologically treated zebrafish. Rapamycin was administered by intraperitoneal injection (5 mg/kg body weight) for a total of nine doses. As control, zebrafish were injected with 5% DMSO in Cortland-solution. To trigger autophagosome accumulation, zebrafish were kept in water containing 1 mM CQ for 3 h before euthanasia. Pooled protein lysates were prepared from 2-3 ventricles and considered as one biological replicate in the analysis. Signal intensities were digitally quantified and then normalized to the respective loading control (GAPDH).

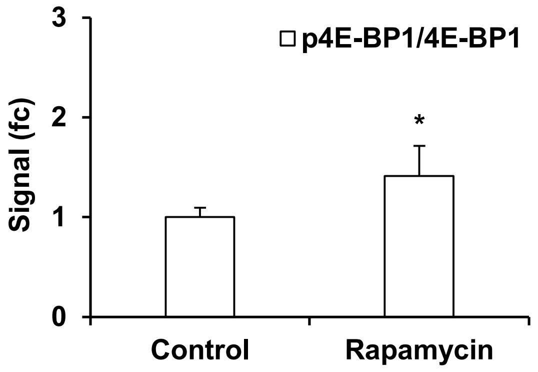

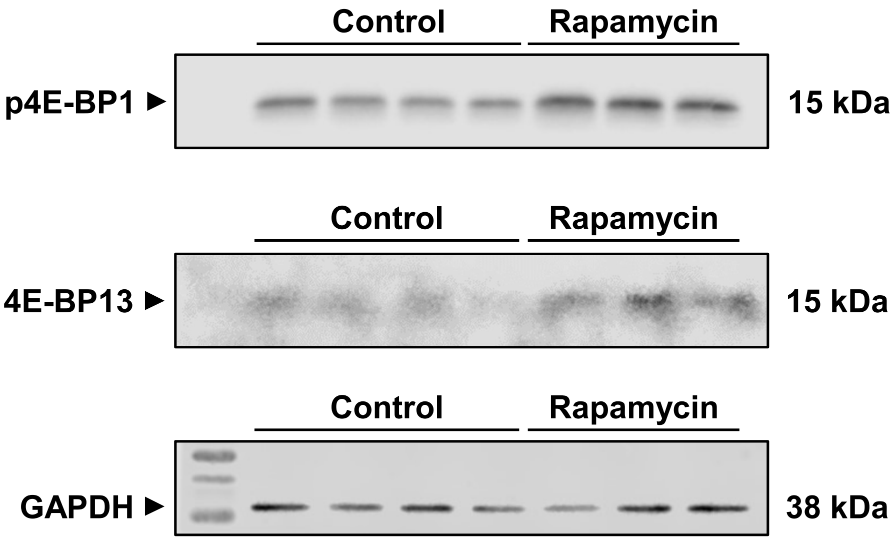

Supp. Fig. 4: Analysis of mTOR-signaling in pharmacologically treated zebrafish. Rapamycin was administered by intraperitoneal injection (5 mg/kg body weight) for a total of nine doses. As control, zebrafish were injected with 5% DMSO in Cortland-solution. To trigger autophagosome accumulation, zebrafish were kept in water containing 1 mM CQ for 3 h before euthanasia. Pooled protein lysates were prepared from 2-3 ventricles and considered as one biological replicate in the analysis. Signal intensities were digitally quantified and then normalized to the respective loading control (GAPDH). Finally, a ratio between the phosphorylated and unphosphorylated form of 4E-BP1 was calculated.

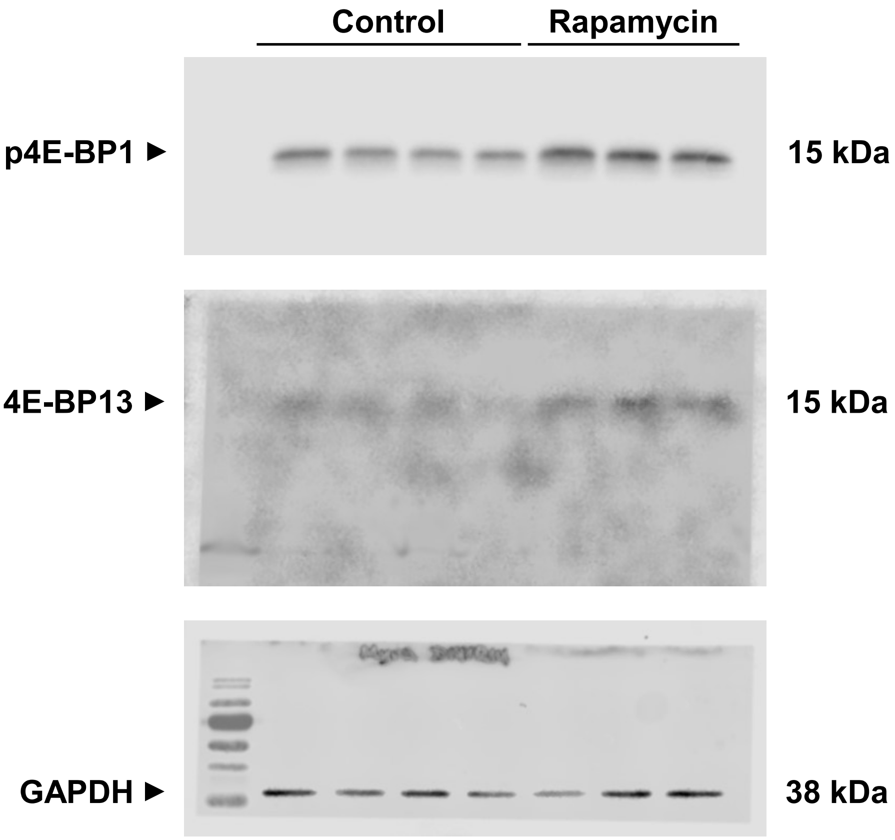

Supp. Fig. 5: Full-length blots used for the analysis of mTOR-signaling in pharmacologically treated zebrafish.
